# The 15th International Conference on Human Retroviruses: HTLV and Related Viruses

**DOI:** 10.1186/1742-4690-8-S1-A1

**Published:** 2011-06-06

**Authors:** Annemie Vandamme, Luc Willems

**Affiliations:** 1 Laboratory of Clinical and Epidemiological Virology, Rega Institute for Medical Research K.U. Leuven, Leuven, Belgium; 2 Cellular and Molecular Biology, Gembloux Agro-Bio Tech, University of Liège, Gembloux, Belgium; 3 Cellular and Molecular Epigenetics, Interdisciplinary Cluster for Applied Genoproteomics, University of Liège, Liège, Belgium

## 

The 15th International Conference on Human Retrovirology: HTLV and Related Retroviruses was organized in Belgium in the cities of Leuven and Gembloux from June 5 to June 8, 2011. The main topics of the meeting were human T-lymphotropic virus (HTLV), Xenotropic murine leukemia virus-related virus (XMRV) and endogenous retroviruses. The following abstracts were presented.

